# Impact of ^18^F-FDG PET/CT on target volume delineation in recurrent or residual gynaecologic carcinoma

**DOI:** 10.1186/1748-717X-7-176

**Published:** 2012-10-22

**Authors:** Hansjörg Vees, Nathalie Casanova, Thomas Zilli, Hestia Imperiano, Osman Ratib, Youri Popowski, Hui Wang, Habib Zaidi, Raymond Miralbell

**Affiliations:** 1 Division of Radiation Oncology, Geneva University Hospital, Geneva 14 CH-1211, Switzerland; 2 Division of Nuclear Medicine and Molecular Imaging, Geneva University Hospital, Geneva, 1211, Switzerland

**Keywords:** Gynaecologic cancer, PET/CT, Radiotherapy, Target volume delineation, Observer variability

## Abstract

**Background:**

To evaluate the impact of ^18^F-FDG PET/CT on target volume delineation in gynaecological cancer.

**Methods:**

F-FDG PET/CT based RT treatment planning was performed in 10 patients with locally recurrent (n = 5) or post-surgical residual gynaecological cancer (n = 5). The gross tumor volume (GTV) was defined by 4 experienced radiation oncologists first using contrast enhanced CT (GTV_CT_) and secondly using the fused ^18^F-FDG PET/CT datasets (GTV_PET/CT_). In addition, the GTV was delineated using the signal-to-background (SBR) ratio-based adaptive thresholding technique (GTV_SBR_). Overlap analysis were conducted to assess geographic mismatches between the GTVs delineated using the different techniques. Inter- and intra-observer variability were also assessed.

**Results:**

The mean GTV_CT_ (43.65 cm^3^) was larger than the mean GTV_PET/CT_ (33.06 cm^3^), p = 0.02. In 6 patients, GTV_PET/CT_ added substantial tumor extension outside the GTV_CT_ even though 90.4% of the GTV_PET/CT_ was included in the GTV_CT_ and 30.2% of the GTV_CT_ was found outside the GTV_PET/CT_. The inter- and intra-observer variability was not significantly reduced with the inclusion of ^18^F-FDG PET imaging (p = 0.23 and p = 0.18, respectively). The GTV_SBR_ was smaller than GTV_CT_ p ≤ 0.005 and GTV_PET/CT_ p ≤ 0.005.

**Conclusions:**

The use of ^18^F-FDG PET/CT images for target volume delineation of recurrent or post-surgical residual gynaecological cancer alters the GTV in the majority of patients compared to standard CT-definition. The use of SBR-based auto-delineation showed significantly smaller GTVs. The use of PET/CT based target volume delineation may improve the accuracy of RT treatment planning in gynaecologic cancer.

## Background

Ultrasound, computed tomography (CT) and magnetic resonance imaging (MRI) are widely recommended in the diagnosis of gynaecologic cancer. These conventional imaging modalities present a high sensitivity, specificity and accuracy in the primary staging of the disease. However, the accuracy and specificity of these techniques for the detection of pelvic tumor recurrences or postsurgical residual disease remains low owing to limitations in distinguishing disease from postsurgical changes [1,2]. CT and MRI may be used for target volume delineation in RT treatment planning of gynaecologic carcinomas. However, a reliable definition of tumor extension is difficult to assess with either modality, especially after surgery. Recently, 18 fluorodeoxyglucose (^18^F-FDG) positron emission tomography – computed tomography (PET/CT) has been recognized as a valuable tool for the diagnosis of primary and recurrent gynaecological cancer enabling the optimization of RT treatment planning [3,4].

The objective of this study is to assess the role of ^18^F-FDG PET/CT based target volume delineation in recurrent or post-surgical residual gynaecologic cancer. We compared the gross tumor volume (GTV) defined manually by four experienced radiation oncologists using contrast-enhanced CT and fused ^18^F-FDG PET/CT images, as well as the biological target volumes (BTVs) defined on the PET/CT semi-automated delineation technique. In addition, we evaluated the inter- and intra-observer variability in the GTV delineation using the above mentioned methods.

## Methods

### Patients

This prospective study was approved by the institutional ethical committee. A signed informed consent was obtained from all patients participating in the study protocol. Between September 2006 and December 2008, 10 patients with a histologically proven local recurrent (n = 5) or post-surgical residual (n = 5) gynaecological cancer were included. Patients didn’t show any evidence of lymph node or distant metastases. Local recurrences were observed at a median of 34 months (range, 9-62 months) after surgery in 4 patients and following postsurgical radio-chemotherapy in 1 patient. The median age was 64 years (range, 40-81 years). The clinical characteristics and referral patterns of the patient population are summarized in Table 1.

**Table 1:** Tumor characteristics and CT- and PET/CT-based GTVs (gross tumor volumes)

**Pat. No.**	**Primary Malignancy**	**FIGO stage**	**Treatment indication**	**GTV**_**CT1**_	**GTV**_**CT2**_	**GTV**_**PET/CT1**_	**GTV**_**PET/CT2**_	**GTV**_**SBR**_
				**Mean (cm**^**3**^**) (SD)**	**Mean (cm**^**3**^**) (SD)**	**Mean (cm**^**3**^**) (SD)**	**Mean (cm**^**3**^**) (SD)**	**(cm**^**3**^**)**
1	Cervix	IIIA	Primary	36.06	36.04	43.28	43.26	25.4
				(4.27)	(6.96)	(13.20)	(10.40)	
2	Cervix	IIB	Primary	60.14	54.88	34.11	27.67	18.3
				(6.60)	(7.03)	(5.45)	(6.80)	
3	Cervix	IIIB	Primary	66.43	95.06	41.69	48.84	22.0
				(14.16)	(10.46)	(2.07)	(1.94)	
4	Uterus	IC	Primary	2.73	4.32	1.91	1.38	1.2
				(0.57)	(2.04)	(0.43)	(0.73)	
5	Vagina	IIIB	Primary	21.48	22.72	12.19	12.33	4.6
				(2.25)	(5.03)	(2.02)	(1.91)	
6	Cervix	IB	Local Recurrence	95.99	96.05	86.74	70.11	67.8
				(9.81)	(19.11)	(3.03)	(12.86)	
7	Vulva	IIIB	Local Recurrence	16.99	15.10	11.23	7.59	5.4
				(5.03)	(5.99)	(2.67)	(0.54)	
8	Uterus	IIIA	Local recurrence	6.39	4.59	3.19	2.24	1.3
				(1.42)	(1.54)	(0.99)	(0.42)	
9	Uterus	IVA	Local Recurrence	26.41	36.45	18.21	15.44	12.7
				(2.54)	(6.16)	(5.37)	(5.93)	
10	Uterus	IIIA	Local Recurrence	103.84	106.11	78.06	98.90	54.6
				(1.72)	(12.20)	(17.14)	(2.99)	

*SBR* = Signal-to-background ratio.

### ^18^F-FDG PET/CT

All 10 patients underwent a diagnostic whole body ^18^F-FDG PET/CT scan performed in treatment planning conditions on the Biograph 16 PET/CT scanner, Siemens Healthcare, Erlangen, Germany. Patients fasted at least 6 hours prior to the start of the examination. A forced-diuresis protocol was used in all patients for a better differentiation between the tumor and the bladder. Thirty minutes after the ^18^F-FDG-injection, each patient received 0.5 mg of furosemide per kilogram of body weight (maximum, 40 mg) followed by infusion of 500 mL of physiologic saline through an intravenous line. One hour after ^18^F-FDG injection and directly after voiding of the bladder, patients were placed in scanning position.

First, a topogram was obtained from the skull to the upper region of the legs. Secondly, ^18^F-FDG PET data were acquired in 3 to 4 minutes bed positions (total of 6 to 7 bed positions) following a low dose CT scan using for attenuation correction. A diagnostic quality contrast enhanced CT scan was then performed.

^18^F-FDG PET, CT and fused ^18^F-FDG PET/CT images were displayed for reviewing axial, coronal, and sagittal planes. All studies were interpreted and reviewed with knowledge of the patient’s clinical history and results of previous imaging studies including MRI of the pelvis in all patients. A combined team of an experienced nuclear medicine physician and an experienced radiologist interpreted the ^18^F-FDG PET/CT images. A multimodality computer platform (Syngo Multimodality Workplace, Siemens Healthcare, Erlangen, Germany) was used for image review and interpretation. All ^18^F-FDG PET/CT studies showing at least one site of abnormal ^18^F-FDG uptake were characterized as malignant. Foci of increased ^18^F-FDG uptake, with intensity higher than that of surrounding tissues, in areas unrelated to physiologic or benign processes, were defined as malignant. Tumor uptake of all lesions was assessed quantitatively using maximum standardized uptake value (SUV) derived by placing a region of interest encompassing the tumor on each slice of transaxial plane.

### Manual contouring protocol

Four experienced radiation oncologists were asked to delineate the GTVs on axial slices of the CT (GTV_CT_) and the ^18^F-FDG PET/CT (GTV_PET/CT_), respectively. Recent T2-weighted contrast enhanced MRI images were also available as additional information for contouring and for fusion on Syngo multimodality software (Siemens Healthcare, Erlangen, Germany). All scans were contoured with knowledge of the additional diagnostic images and reports.

The contouring process consists of the following steps: firstly the radiation oncologists delineated the GTV on the contrast-enhanced CT images alone (GTV_CT1_). The images and reports of the ^18^F-FDG PET were blinded. Then, after at least two weeks the observers contoured the BTV on the fused ^18^F-FDG PET/CT images (GTV_PET/CT1_). To assess the intra-observer variability, all observers were asked to contour the target volume a second time two months later on CT images (GTV_CT2_) and once again two weeks later on the ^18^F-FDG PET/CT images (GTV_PET/CT2_). They were blinded to their previous contours as well as to those of the other observers. The radiation oncologists were all trained in target volume delineation on PET/CT and were free to adjust the window, level and contrast setting of the images.

### Signal-to-background ratio-based (SBR) adaptive thresholding (GTV_SBR_)

For GTV_SBR_ delineation, the maximum signal intensity of the tumor was defined as the mean activity of the hottest voxel and its eight surrounding voxels in a transversal slice, whereas the mean background activity was obtained from a manually drawn ROI far away from the tumor [5]. The SBR-thresholding technique has been described in a previous publication by our group [6]. The GTV_SBR_ were checked visually before approval.

### Contour analysis

The delineated contours for both delineation phases were analyzed separately. Firstly, the volumes contoured by every observer for GTV_CT_ and GTV_PET/CT_ were calculated for every patient separately and the composite and common volume of GTV_CT_ and GTV_PET/CT_ were calculated. The composite volume PET/CT is the sum of GTV_CT1_ and GTV_PET/CT1_ while the common volume PET/CT is the joint volume of GTV_CT1_ and GTV_PET/CT1_ of each observer. To assess the geographic mismatch between the GTVs delineated using the different segmentation techniques, the following overlap analyses were performed: (A) The overlap volume of GTV_CT1_ and GTV_PET/CT1_, for which overlap was expressed as the overlap volume of GTV_CT1_ and GTV_PET/CT1_ relative to the CT-based GTVs − overlap fraction (OF) CT_1_ [OF_CT1_]; (B) the OF of GTV_PET/CT1_ and GTV_CT1_ relative to the PET/CT-based GTV − overlap fraction PET/CT1 [OF_PET/CT1_]. In addition, the overlap volume of GTV_PET/CT1_ and GTV_SBR_ relative to GTV_SBR_-OF was also calculated [OF_SBR_] (C).

Inter- and intra-observer variability was calculated using a two-way ANOVA model. Regression analysis was used to evaluate the difference between calculated volumes and overlap between GTVs when using the different segmentation tools. Statistical analysis and curve fitting was performed using PASW Statistics package, version 18.0 (IBM, Chicago, Illinois, USA). The level of statistical significance adopted was 0.05.

## Results

**Figure 1:**
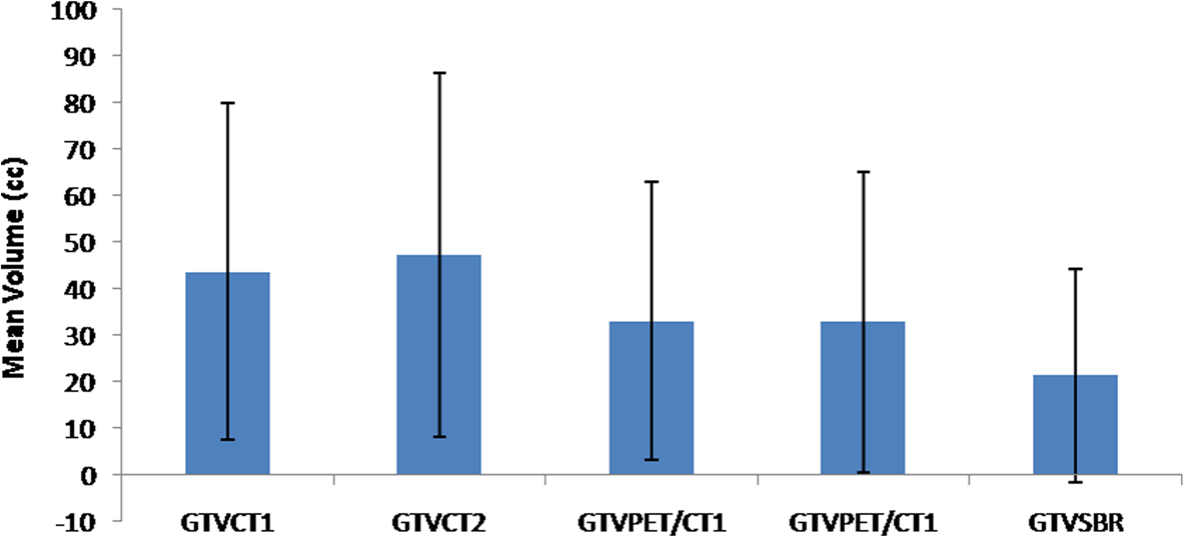
**Comparison of mean tumor volumes using the different manual and SBR delineation techniques. **Error bars indicate standard deviation (SD) on the mean. Results are shown for the gross tumor volume (GTV) delineated on CT (GTV_CT1_ and GTV_CT2_) and PET/CT-based GTVs obtained by manual delineation of contours (GTV_PET/CT1_ and GTV_PET/CT2_), and signal-to-background ratio (SBR)-based adaptive thresholding (GTV_SBR_).

**Figure 2:**
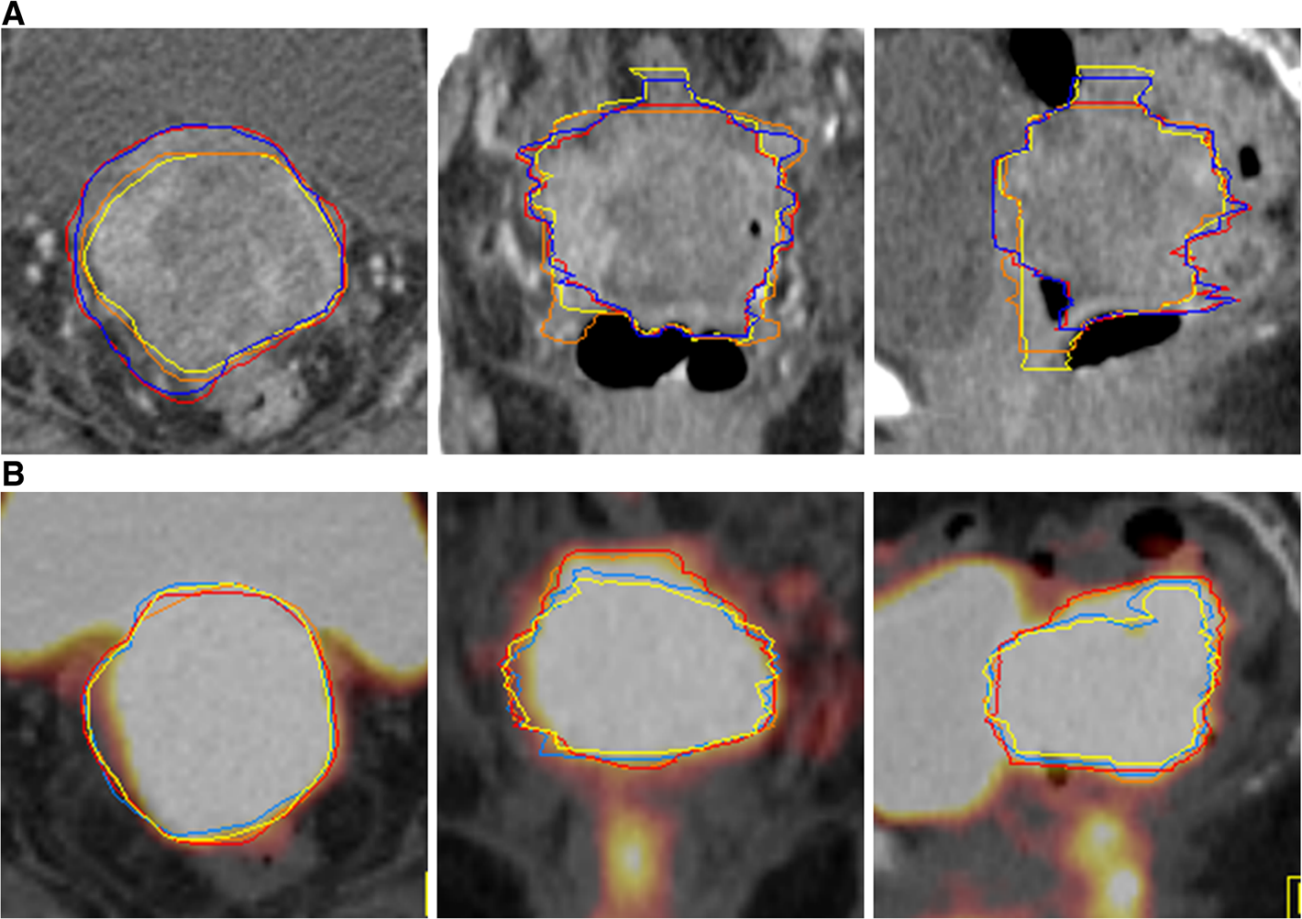
^18^**F-FDG PET with coregistered contrast enhanced CT showed a local recurrence with a SUV**_**max **_**of 16.16 in a 60 year old patient 6 months after total hysterectomy, adnexectomy and pelvic lymphadenectomy for an endometrial cancer FIGO IIIA. (A)** GTV_CTs_ defined by four observers on axial, sagittal and coronal contrast enhanced CT. (**B**) GTV_PET/CTs_ defined by four observers on axial, sagittal and coronal ^18^F-FDG PET/CT. In panel B we observed a greater interobserver agreement and the GTV_PET/CTs_ were smaller than the GTV_CTs_.

The contrast enhanced CT scan as well as the ^18^F-FDG PET/CT were able to pinpoint the local recurrent or residual cancer in the pelvis. The median SUV_max_ of GTVs was 11.74 (range, 7.55 -17.82). We did not observe any difference in PET signal between residual tumor and recurrent tumors. Figure 1 presents the mean tumor volumes using the different manual and SBR delineation techniques. Error bars indicate standard deviation (SD) on the mean. Wide variability of the GTV_CT_ and GTV_PET/CT_ was observed. The mean GTV_CT1_ (43.65 cm^3^, SD 4.84) was significantly larger than the mean GTV_PET/CT1_ (33.06 cm^3^, SD 5.24), p = 0.02. The smallest GTV_CT1_ and GTV_PET/CT1_ was found in patient #6 with 1.89 cm^3^ and 0.85 cm^3^ respectively, and the largest GTV_CT_ in patient #4 with 120.39cm^3^, while the largest GTV_PET/CT_ was observed in patient #10 (101.93 cm^3^). Figure 2 presents an example of the GTVs contoured by each observer in each modality in a patient with a local recurrent cervical cancer. The contouring of this case was hampered by the adjacent localization of the bladder and the rectum.

Table 2 summarizes the comparative evaluation of the CT- and PET/CT-based GTVs. The mean composite volume was 46.15 cm^3^ (SD 5.42) and the mean common volume was 31.48 cm^3^ (SD 4.21). The mean OF_CT1_ was 0.63 (SD 0.04). The mean OF_PET/CT1_ was 0.90 (SD 0.03). In 2 patients, the GTV_PET/CT_ of all observers was included entirely in the GTV_CT_ and in 6 patients, GTV_PET/CT_ added substantial tumor extension outside the GTV_CT_.

**Table 2:** **Summary of the composite and common volumes of GTV**_**CT1 **_**and GTV**_**PET/CT1 **_**as well as overlap fractions (OFs) between the GTV**_**CT1**_**, GTV **_**PET/CT1 **_**and GTV**_**SBR**_

**Pat. No.**	**Composite volume PET/CT**	**Common volume PET/CT**	**OF**_**CT1**_	**OF**_**PET/CT1**_	**OF**_**SBR**_
	**Mean (cm**^**3**^**) (SD)**	**Mean (cm**^**3**^**) (SD)**	**Mean (cm**^**3**^**) (SD)**	**Mean (cm**^**3**^**) (SD)**	**Mean (cm**^**3**^**) (SD)**
1	6.95	5.02	0.72	0.91	0.96
	(SD 1.32)	(SD 0.95)	(SD 0.03)	(SD 0.04)	(SD 0.04)
2	47.24	46.73	0.55	0.81	1.00
	(SD 11.80)	(SD 5.43)	(SD 0.04)	(SD 0.04)	(SD 0.00)
3	64.82	44.56	0.59	0.93	1.00
	(SD 6.02)	(SD 13.68)	(SD 0.04)	(SD 0.02)	(SD 0.00)
4	95.99	99.61	0.57	1.00	1.00
	(SD 8.01)	(SD 1.63)	(SD 0.05)	(SD 0.00)	(SD 0.00)
5	20.35	20.82	0.45	0.81	0.91
	(SD 6.53)	(SD 4.45)	(SD 0.03)	(SD 0.03)	(SD 0.08)
6	2.81	2.43	0.77	0.98	1.00
	(SD 0.75)	(SD 0.62)	(SD 0.03)	(SD 0.03)	(SD 0.00)
7	26.16	12.89	0.61	0.85	0.90
	(SD 1.99)	(SD 0.94)	(SD 0.06)	(SD 0.06)	(SD 0.05)
8	66.43	50.11	0.46	1.00	1.00
	(SD 14.08)	(SD 2.31)	(SD 0.03)	(SD 0.00)	(SD 0.00)
9	27.64	19.48	0.67	0.96	1.00
	(SD 0.94)	(SD 2.78)	(SD 0.03)	(SD 0.04)	(SD 0.00)
10	105.06	97.49	0.87	0.79	0.97
	(SD 2.74)	(SD 9.30)	(SD 0.10)	(SD 0.02)	(SD 0.03)

The composite volume PET/CT is the sum of GTV_CT1_ and GTV_PET/CT1_ while the common volume PET/CT is the joint volume of GTV_CT1_ and GTV_PET/CT1_ of each observer. [OF_CT1_] is the overlap fraction (OF) of GTV_PET/CT1_ relative to GTV_CT1_ while [OF_PET/CT1_] is the OF of GTV_CT1_ relative to GTV_PET/CT1_ and [OF_SBR_] is the OF of GTV_PET/CT1_ relative to GTV_SBR_. SBR = Signal-to-background ratio.

We found that among four experienced radiation oncologists, the ratio of largest to smallest GTVs outlined on 10 patients using the planning CT had a median of 1.87 (range, 1.21 to 3.27). When the ^18^F-FDG-PET was included, this ratio was reduced to median 1.38 (range, 1.16 to 1.81). The ratio of largest to smallest GTV was decreased in 9 of 10 patients using PET/CT for GTV delineation.

### Evaluation of inter- and intra-observer variation

The median inter-observer reliability index for the GTV_CT_ was 0.37 (range, 0.21-0.63) and for the GTV_PET/CT_ was 0.48 (range, 0.32-0.71); p = 0.23. All physicians contoured each patient twice and the median intra-observer percentage of concordance for the GTV_CT_ was 0.49 (range, 0.13-0.89) and for the GTV_PET/CT_ was 0.65 (range, 0.30-0.92) (p = 0.18).

### SBR-based auto-contour compared with manual delineation

The GTVs were delineated both manually and by editing the SBR-based auto-contour. The results concerning GTV_SBR_ are shown in Table 1. The mean GTV_SBR_ was 21.33 cm^3^ (SD 23.87), which is significantly smaller than the manually contoured GTV_CT_ (p ≤ 0.005) and GTV_PET/CT_ (p ≤ 0.005). In 6 patients the GTV_SBR_ was included completely in all GTV_CTs_ and the mean OF between GTV_SBR_ and GTV_PET/CT_ was 0.97 (SD 0.02). Comparing the GTV_SBR_ with the GTV_PET/CTs_, we observe that in 4 patients the GTV_SBR_ were larger than the GTV_PET/CT_.

## Discussion

CT and MRI have reasonable sensitivity but low specificity in identifying recurrent gynaecologic disease [1,2]. Consequently, significant observer variation has been noted in contouring the GTV_CT_[7]. ^18^F-FDG PET/CT plays an increasingly important role in the staging and management of gynaecologic cancer including RT treatment planning [3,4]. ^18^F-FDG PET/CT has demonstrated a high sensitivity and accuracy of more than 90% with average specificity in locally advanced or recurrent gynaecologic pelvic carcinoma. Furthermore ^18^F-FDG PET/CT can help to distinguish between tumor recurrence and post-therapy changes [4,8]. Kidd et al. have shown that cervical cancer patients treated with ^18^F-FDG PET/CT-guided IMRT had improved survival and decreased treatment related toxicity compared with patients treated with non-IMRT radiotherapy [9].

This delineation study evaluated inter- and intra-observer variability of CT-based and ^18^F-FDG PET/CT-based target volume delineation in local recurrent or postsurgical residual gynaecological cancer. The results were compared with an automated PET segmented technique using adaptive thresholding technique. In other cancer sites such as head and neck and lung, ^18^F-FDG PET/CT was reported to decrease inter- and intra-observer variability in tumor contouring [10]. Our results suggest that GTV delineation using ^18^F-FDG PET/CT could be superior to CT alone in this group of patients. GTV_PET/CT_ was significantly smaller than the GTV_CT_ with a trend for reduced inter- and intra-observer variability using PET/CT. The inter-observer agreement was moderate for the GTV_CT_ and substantial for the GTV_PET/CT_[11]. The inter-observer reliability was lower than the intra-observer reliability. This is in agreement with observations made by other authors [12]. It has been considered that the observers tend to agree more with themselves rather than with each other. Inter- and intra-observer variability has been mostly investigated in lung cancer and the increased observer reliability on ^18^F-FDG PET/CT in our study is in line with these findings [10]. Only one study by our group evaluated the inter-observer variability in PET/CT-based target volume delineation in the pelvis [13]. A trend of reduced inter-observer variability has been observed in the delineation of the intraprostatic recurrence lesion using ^18^F-choline PET/CT. In gynaecologic cancer no inter- or intra-observer variability in PET-based GTV-delineation has been evaluated until now.

Our study demonstrate that the size of GTV_PET/CT_ was significantly smaller than the GTV_CT_ with the implementation of a coregistered ^18^F-FDG PET/CT_._ When the GTV_SBR_ volumes were analyzed and compared with manual delineated target volume, it was observed that the GTV_SBR_ was significantly smaller than the median GTV_CT_ and GTV_PET/CT_. This was also manifested in the overlap analysis, where the overlap fraction increased from OF_CT1_ to OF_PET/CT1_ and OF_SBR_. Overall, the comparison of GTVs delineated in primary and recurrent cancer did not result in any significant differences.

The strength of our study includes the use of contrast enhanced CT scans for GT_CT_ and GTV_PET/CT_ determination and that the exams were performed on a dedicated PET/CT scanner for virtual simulation and fused with a recent MRI. Nevertheless the inter- and intra-observer variability was relatively high with both imaging modalities, highlighting the difficulty to determine the target volumes in this group of patients. An automated segmentation of the target volume using the adaptive thresholding technique could eventually help to reduce inter- and intra-observer variability. One potential limitation of our study is that the observers were at liberty to adjust the window, level and contrast setting of the images. This could have increased the inter- and intra-observer variability. However, all observers were experienced in PET/CT-based target volume delineation and were helped by a nuclear medicine physician. Another drawback of this study is the lack of comparison of the PET/CT results with pathologic findings after surgery.

The delineation of target volumes and organ at risk is a very critical step in high-precision RT treatment planning. Good image quality and reliable delineation protocol are important for accurate target volume delineation. One of the challenges of PET/CT-guided target volume delineation is the accurate segmentation of noisy and low resolution functional PET images. This is in particular true in recurrent or residual gynaecological cancer where vascular and urinary activity hampers target volume delineation. The result is a relatively high inter- and intra-observer variability. Various PET image segmentation techniques for target volume delineation were developed and evaluated to overcome this drawback [11]. Among them, manual contouring by visual examination is the most commonly used method. The determination of an appropriate window and level for viewing the PET images is highly operator-dependent and is subject to high variability between operators [12].

An improved concordance in target volume delineation using PET/CT implies a greater accuracy and can help to determine a more appropriate treatment plan. In our study, the inter-observer variability coefficient prevailed is still relatively high. Variability negatively impacts the quality of treatments delivered to cancer patients. Alternatively an automated segmented target volume could be considered. There is consensus in the need for highly objective and automatic segmentation methods, and various groups have observed that semi- or fully-automated delineation techniques reduce inter-observer variability and improve reproducibility [10]. The adaptive thresholding technique is one of the most widely used segmentation techniques for target volume determination in clinical setting. However, knowledge of the true target volume in relation the GTV_SBR_ in gynaecologic tumors is needed for validation purposes. PET-based target volume delineation in gynaecologic tumors is actually not recommended outside clinical studies. It has to be emphasized that both patients with recurrent or postsurgical residual gynaecologic cancer are challenging cohorts for reliable target volume delineation and thus it is more likely that high inter- and intra-observer variability will result. In the absence of a more accurate information on the target volume position in gynaecologic cancer, a composite of GTV_CT_ and GTV_PET/CT_ can be recommended to optimize the GTV definition.

## Conclusions

This delineation study showed that GTV_PET/CT_ was significantly smaller than GTV_CT_. The reduction was larger when the adaptive thresholding-based semi-automated contouring algorithm was used. GTV_PET/CT_ added substantial tumor extension outside the GTV_CT_ in 60% of the patients. The combination of a matched ^18^F-FDG PET/CT reduced the inter- and intra-observer variation in the delineation of gynaecological cancer however the difference was not significant. Target volume delineation may be improved with the inclusion of ^18^F-FDG PET/CT.

## Competing interests

The authors declare that they have no competing interests.

## Authors’ contributions

HV carried out the drafting of the manuscript and partially the analysis of the data. RM carried out the design of the study. HW and NC defined the target volumes and participated in the analysis of the data. TZ defined the target volumes and participated in the analysis of the data. YP participated in the target volume definition. HI performed the imaging analysis. HZ participated in the analysis of the data. OR participated in the methodological design. All authors read and approved the final manuscript.
